# Karnofsky Performance Score—Failure to Thrive as a Frailty Proxy?

**DOI:** 10.1097/TXD.0000000000001164

**Published:** 2021-06-08

**Authors:** Margaret R. Stedman, Daniel J. Watford, Glenn M. Chertow, Jane C. Tan

**Affiliations:** 1 Department of Medicine, Division of Nephrology, Stanford University School of Medicine, Palo Alto, CA.

## Abstract

Among patients listed for kidney transplantation, the Karnofsky Performance Status (KPS) Scale has been used as a proxy for frailty and proposed as a predictor of long-term posttransplant outcomes. The KPS is required by the Organ Procurement and Transplantation Network for all transplants; however, the interrater reliability of KPS reporting in kidney transplant candidates has not been well investigated, and there is concern regarding limitations of using KPS that may influence transplant eligibility.

**Methods.:**

We performed an observational study using existing Scientific Registry of Transplant Recipients data from 2006 to 2020 to examine the variability, reliability, and trends in the KPS among patients on the kidney transplant waitlist.

**Results.:**

Our analysis included 8197 kidney transplant candidates with >1 KPS in a 3-mo period. We observed 2–7 scores per patient with an average score of 78.9 (SD = 12, 95% confidence interval, 78.8-79.1). We found substantial variability in KPS reporting, in which 27% of the patients had scores that varied widely with 20–80 points in difference. Interrater reliability in the 10-point scale was poor (30%). When using a condensed 4-category scale (disabled, requires assistance, capable of self-care, normal activity), 38% of patients experienced at least a 1-category shift in their score.

**Conclusions.:**

The lack of reliability in KPS reporting raises concerns when applying the KPS as a proxy for frailty and a metric to be considered when evaluating candidacy for kidney transplantation.

## Abstract 2

Supplemental Digital Content is available in the text.

## INTRODUCTION

Frailty is a pathobiological process characterized by loss of physiologic reserve and increased vulnerability to stressors.^1^ Frailty portends worse outcomes in solid organ transplantation.^2^ Although there is increasing interest in incorporating frailty measures in solid organ transplantation, no standardized measures of frailty (eg, the Fried criteria) have been collected in national registries. Hence, investigators have used proxy metrics to evaluate the implications of frailty in studies of transplant candidates. The Karnofsky Performance Status (KPS) is a subjective measure of functional impairment. In prospective kidney transplant candidates, KPS is collected by a healthcare provider at the time of listing and repeated at the time of transplant as well as in follow-up visits. These data are required by the Organ Procurement and Transplantation Network (OPTN) for all transplants and until 2011, was used to risk-adjust center-specific outcomes.^3,4^

**FIGURE 1.**
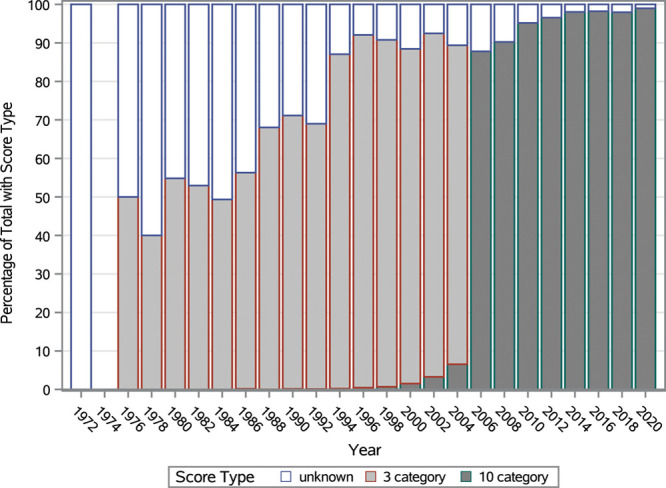
Distribution of Karnofsky Performance Status type by year.

The KPS Scale was developed in 1948 for patients with cancer to measure their functional status and to predict short-term survival.^5^ The scale ranges from normal functioning (100%) to dead (0%) with increments of 10% in between. Each incremental increase describes the level to which the patient is able to perform his or her daily activities independently (see Table 1). For example, a score of 40% describes a patient who is disabled, whereas a score of 60% denotes a patient who requires occasional assistance. In the Scientific Registry of Transplant Recipients (SRTR) data, scores exist back to year 1973. From 1973 to 1985, there was sparse reporting with only 3 categories: normal functioning (80%–100%), unable to work (50%–70%), and unable to care for self (0%–40%). From 1986 to 2005, the KPS evolved into a mix of the 3 categories and the 10-point scale. In 2006, all scores were converted to the 10-point scale, as shown in Figure 1. Among patients listed for transplantation, the KPS has been used as a proxy for frailty and proposed as a predictor of long-term posttransplant outcomes.^6^

**TABLE 1.** Karnofsky Performance Status Scale

0%	Dead
10%	Moribund, fatal processes progressing rapidly
20%	Very sick, hospitalization necessary: active treatment necessary
30%	Severely disabled: hospitalization is indicated, death not imminent
40%	Disabled: requires special care and assistance
50%	Requires considerable assistance and frequent medical care
60%	Requires occasional assistance but is able to care for needs
70%	Cares for self: unable to carry on normal activity or active work
80%	Normal activity with effort: some symptoms of disease
90%	Able to carry on normal activity: minor symptoms of disease
100%	Normal, no complaints, no evidence of disease

The reliability of reporting KPS in kidney transplant candidates has not been well studied. Several studies have evaluated the interrater reliability in patients with cancer.^7–9^ They reported moderate to high degrees of correlation among raters, with some increased variability between the type of rater (nurse, physician, social worker). Despite its limitations, the KPS continues to be used as a proxy for frailty and may influence kidney transplant eligibility. In turn, this may translate into poorer access to transplantation in some disadvantaged populations.

To address our concerns regarding reliability and validity of the KPS as a proxy for frailty, we performed an observational study using existing SRTR data to examine the degree of variation in reporting of KPS. Patient listings at multiple sites allows for a natural experiment in which multiple scores are collected by different raters in a relatively short period of time. Using data available from 2006 to 2020, we evaluated the variability, reliability, and trends in the KPS among patients on the kidney transplant waitlist.

## MATERIALS AND METHODS

This study was performed in accordance with the Declaration of Helsinki and was approved by the Stanford Institutional Review Board (protocol no. 40876). The clinical and research activities being reported are consistent with the Principles of the Declaration of Istanbul as outlined in the “Declaration of Istanbul on Organ Trafficking and Transplant Tourism.” Because of the retrospective and observational nature of the research, the need for written informed consent was waived.

### Study Population

This study used data from the SRTR. The SRTR data system includes data on all donors, waitlisted candidates, and transplant recipients in the United States, submitted by the members of the OPTN. The Health Resources and Services Administration, US Department of Health and Human Services, provides oversight to the activities of the OPTN and SRTR contractors. We selected adult (age 18+) patients on the kidney transplant waitlist from January 1, 2006, to June 2, 2020. We opted to exclude listings before 2006 where there was a mix of coding the KPS as 3 categories instead of the 10-point scale used today. We selected pretransplant scores only and excluded multiorgan (eg, liver-kidney, pancreas-kidney) candidates. Ideally, we wanted measurements as close in time as possible to reduce the chance for functional decline without losing generalizability across the US sample. In reality, multiple listings require time for referral and travel. To address this, we restricted the analysis to patients with at least 2 scores within 3 mo.

The KPS was categorized into 4 groups: normal activity (80%–100%), capable of self-care (70%), requires assistance (50%–60%), and disabled (10%–40%). The additional category at KPS = 70% was partitioned out to address some of the skewness in the data (see the Sensitivity Analysis section). We included the following covariates: age, sex, race, ethnicity, Quételet (body mass) index (BMI), education, employment status, insurance type, year of listing, diabetes, peripheral vascular disease, chronic obstructive pulmonary disease, cerebrovascular disease, coronary artery disease, blood type, dialysis vintage, and previous transplant. We included patient ID and center ID to adjust for subject-to-subject and center-to-center differences. These variables are all recorded in the transplant candidate’s file within the SRTR database and updated at each time of listing. We specified the KPS and covariates at the time of the first listing as baseline measurements.

### Statistical Methods

We described covariate data by baseline KPS category as frequencies and percentages. We performed a descriptive analysis on the range of scores for each patient by comparing the maximum and minimum scores in the 3-mo period. Because not all patients were evaluated at all the centers and there were variable numbers of measurements per patient, usual kappa tests of interrater agreement could not be performed.^10,11^ Instead we estimated the intraclass correlation coefficient (ICC) from random effects models with patient and center random effects^12^ (see Appendix S1, SDC, http://links.lww.com/TXD/A334). The ICC is an estimate of the intrarater agreement in which <50% is considered poor reliability, 50%–75% is moderate reliability, 76%–90% is good reliability, and 91%–100% is excellent reliability.^13^ We conducted all analyses with SAS for Windows version 9.4,^14^ R Studio v1.3,^15^ and Winbugs v14.^16,17^

## RESULTS

**FIGURE 2.**
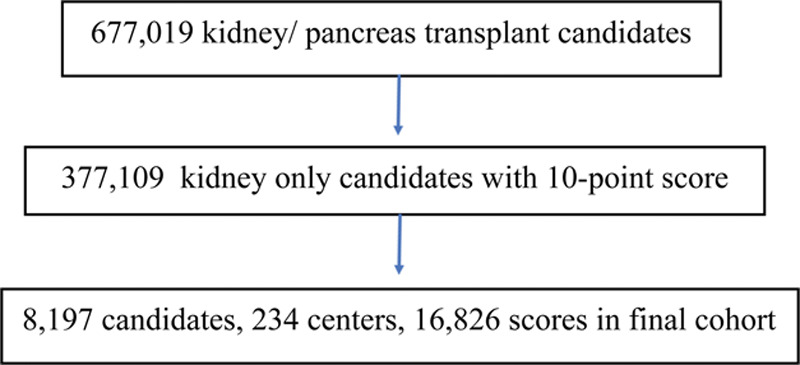
Cohort definition flow diagram.

There were 677 019 adult patients listed for a kidney or pancreas transplant in the SRTR KI/PA candidacy file with a KPS score (1972–2020) and a valid center ID. From these, we excluded patients who were listed for a pancreas transplant along with patients simultaneously listed in the other transplant candidacy lists (lungs, heart, liver, and intestines). We excluded all listings before 2006 and KPS scores that did not fall in 0%–100% range (recorded as 2010–2100 in the SRTR files). After these exclusions, 377 109 (55.7%) remained. Finally, we restricted the cohort to 8197 (2.2%) patients with >1 KPS in a 3-mo period (see Figure 2).

Each patient was categorized by the baseline KPS score as disabled, requires assistance, capable of self-care, or normal activity. The majority (5348 or 65%) of patients had a first KPS in the normal range, whereas only 1% (n = 86) had a KPS in the disabled range. Lower KPS was associated with racial (45% of disabled versus 33% of normal are nonwhite) and ethnic diversity (21% of disabled versus 12% of normal were Latino). We observed a dramatic shift in the KPS by year of observation, where lower KPS tended to be more common in recent years (2016–2020) than historically (47% versus 8% disabled). Patients with low KPS were more likely to be on dialysis and have longer dialysis vintage than patients with a high KPS (17% of disabled versus 6% of normal) (see Table 2).

**TABLE 2.** Patient and center demographics by category of the Karnofsky Performance Status at first listing

DemographicN (%)	All	Disabled10–40	Requires assistance50–60	Capable of self-care70	Normal activity80–100
Patients	N = 8197	N = 86	N = 638	N = 2125	N = 5,348
Age					
18–40 y	1732 (21%)	26 (30%)	132 (21%)	402 (19%)	1172 (22%)
41–64 y	4959 (61%)	54 (63%)	388 (61%)	1306 (62%)	3211 (60%)
65+ y	1506 (18%)	6 (7%)	118 (19%)	417 (20%)	965 (18%)
Female	3056 (37%)	40 (47%)	233 (37%)	834 (39%)	1949 (36%)
Race					
White	5352 (65%)	47 (55%)	397 (62%)	1336 (63%)	3572 (67%)
Asian	696 (9%)	14 (16%)	45 (7%)	141 (7%)	496 (9%)
Black	2057 (25%)	22 (26%)	189 (30%)	619 (29%)	1227 (23%)
Other^*a*^	92 (1%)	3 (4%)	7 (1%)	29 (1%)	53 (1%)
Latino	1213 (15%)	18 (21%)	109 (17%)	424 (20%)	662 (12%)
Year					
2006–2010	2366 (29%)	7 (8%)	174 (27%)	358 (17%)	1827 (34%)
2011–2015	2980 (36%)	39 (45%)	199 (31%)	889 (42%)	1853 (35%)
2016–2020	2851 (35%)	40 (47%)	265 (42%)	878 (41%)	1668 (31%)
Comorbidity					
Diabetes^*b*^	3092 (38%)	36 (42%)	312 (49%)	998 (47%)	1746 (33%)
COPD	42 (<1%)	0 (<1%)	0 (0%)	14 (1%)	28 (1%)
PVD	297 (4%)	6 (7%)	36 (6%)	82 (4%)	173 (3%)
Cerebrovascular disease	74 (<1%)	2 (2%)	9 (1%)	18 (1%)	45 (1%)
Coronary artery disease	208 (3%)	0 (<1%)	17 (3%)	71 (3%)	120 (2%)
Dialysis vintage					
None	2662 (33%)	6 (7%)	102 (16%)	359 (17%)	2195 (41%)
<3 y	4810 (59%)	65 (76%)	461 (72%)	1449 (68%)	2835 (53%)
3+ y	725 (9%)	15 (17%)	75 (12%)	317 (15%)	318 (6%)

^*a*^Other includes multiracial, Native American, and Pacific Islander races.

^*b*^Includes both type I and type II diabetes.

COPD, chronic obstructive pulmonary disease; PVD, peripheral vascular disease.

**FIGURE 3.**
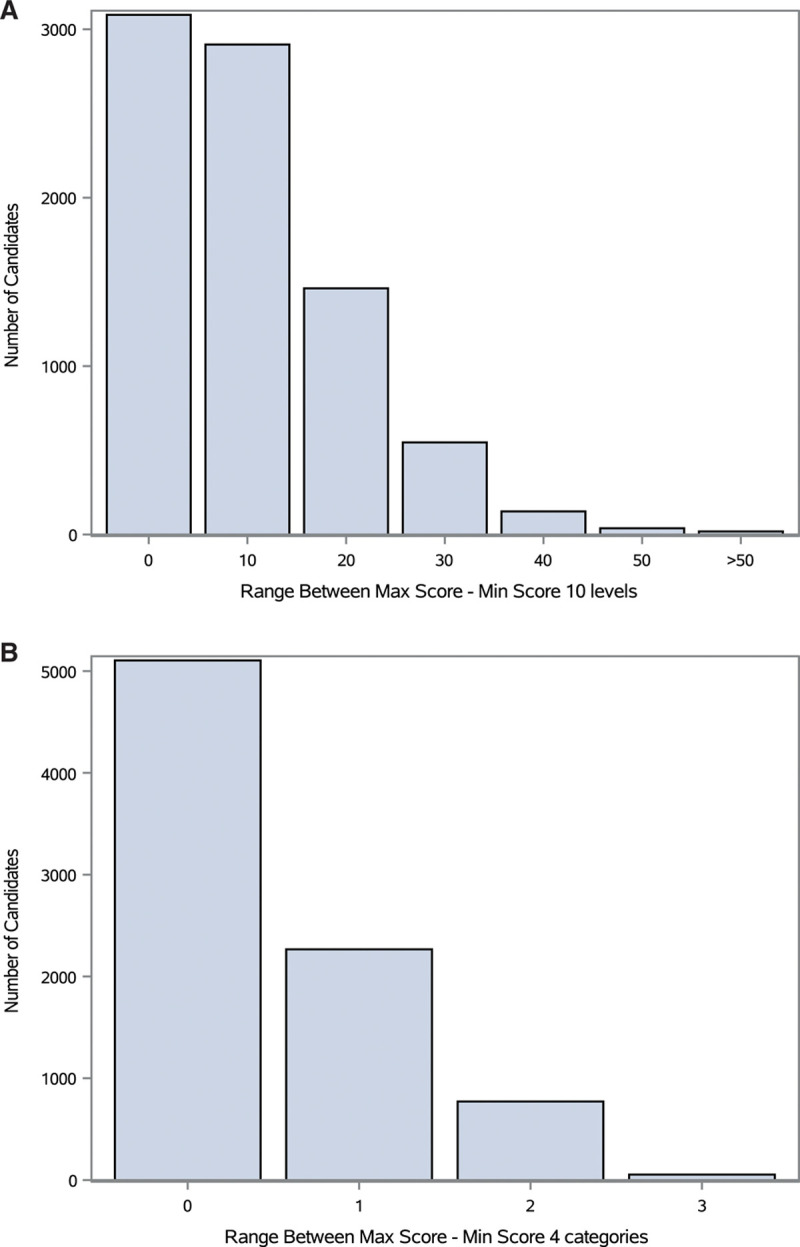
Distribution of the per patient range in Karnofsky Performance Status (left: 10-point score, right: 4-category score).

We observed 2–7 scores per patient in the 3-mo period. The average score was 78.9 (SD = 12; 95% confidence interval, 78.8-79.1). Thirty-eight percent of patients were repeatedly scored the same (ideal), 36% had minimal difference (10 points), whereas the remaining 27% of patients had scores that varied widely with 20–80 points in difference (see Figure 3). For 62% of patients, this represented no difference in their 4-category KPS. For 28% of the patients, this represented a 1-category change in score, whereas the remaining 10% experienced a 2- to 3-category change in the 4-category KPS.

To more rigorously quantify, the variation and reliability of the scores, we applied random effects models to the data (see Table 3). For the 10-point score, the ICC estimate was 30% (poor), and for the 4-category score, the ICC was 43% (poor). With the addition of a center effect to the model, the variance is repartitioned to estimate the correlation between different centers evaluating the same patient (17%, poor) and the correlation between scores from the same center and same patient (17% + 26% = 43%, poor). When we fully adjusted the model, there was a reduction in the total variance of 6% (data not shown). The ICC for the 10-point score conditional on the covariates was 27% (poor). The conditional (fully adjusted) correlations between centers and within centers were 14% (poor) and 41% (14% + 27%, poor).

**TABLE 3.** Percentage of variation explained^a^ by each of the variance components

Unadjusted statistics	10-category scoreMean (SD) = 78.9 (12)Variance = 151.23		4-category scoreMedian (IQR) = 4(1)Variance = 0.5	
Model	Time and year adjusted	Fully adjusted^*b*^	Time and year adjusted	Fully adjusted^*b*^
Patient only random effects model Patient ICC (95% CI)	30% (28%-32%)	23% (21%-25%)	43% (41%-46%)	36% (33%-39%)
Patient + center random effects model Patient ICC (95% CI)Center ICC (95% CI)	17% (15%-19%) 26% (22%-30%)	14% (12%-15%) 27% (23%-31%)	Could not be estimated	Could not be estimated

^a^Percentage of variation explained by each variance component is measured by the ICC. Note that the total variance in KPS will vary by model as different variables are included.

^b^Adjusted for age, sex, race, ethnicity, comorbidity, dialysis vintage, time, and year.

CI, confidence interval; ICC, intraclass correlation coefficient; IQR, interquartile range; KPS, Karnofsky Performance Status.

### Sensitivity Analysis of Model Assumptions

SAS software for analyzing nonlinear random effects models is currently limited to a single random effect. To check the fit of the linear random effects model to the KPS data, we compared results against a nonlinear random effects model assuming an ordinal response. Data were sparce at the low end of the score; thus, we collapsed scores in the 10–30 range to make an 8-category score (<30, 40, 50, 60, 70, 80, 90, 100) that could be directly compared between models. ICC estimates were similar for the 8-category model, suggesting slight bias in the estimate of the ICC. When the data were reduced to a 4-category score, the linear random effects model underestimated the ICC by a difference of 13% (see Appendix S2, Table S1, SDC, http://links.lww.com/TXD/A334). Therefore, we only reported the nonlinear random effects model results in Table 3 for the 4-category data.

We compared results between the 3-category score used historically^18,19^ to the 4-category score specified in this analysis (see Appendix S2, Table S1, SDC, http://links.lww.com/TXD/A334). The ICC for the 3-category score was 54% (moderate agreement) compared with 43% (poor agreement) for the 4-category score.

### Sensitivity to Selection Bias

To test for selection bias in our cohort, we expanded the 3-mo criteria in the cohort definition to include patients with >1 visit in 6 mo and in 1 y (see Appendix S3, Table S2, SDC, http://links.lww.com/TXD/A334). Increasing the time window by 3 mo reduced the reliability of the KPS by 5%.

## DISCUSSION

We sought to estimate the reliability and variability of the KPS in a national sample using the SRTR data. We took advantage of the process of multiple transplant program listing to obtain independent assessments of the KPS. We found substantial variability in KPS reporting and a poor interobserver reliability of 30% in the 10-point scale. More than 1 in 4 (27%) patients experienced more than a 10-point shift in KPS using the 100-point (10-category) scale, while 38% experienced at least a 1-category shift when using a condensed 4-category scale. The ICC for the 4-category score could also be considered an estimate of the consistency between measures because it allows for more error than the 10-point scale.^13^ Although the condensed 4-category scale improved reliability of the instrument, there is also some loss of information in reducing the scale from 10 to 4 to 3 categories. Fifty-four percent of agreement (moderate) for the 3-category score represents a significant gain in improvement over the ICC estimate of 43% (poor agreement) for the 4-category score and the estimate of 30% (poor agreement) for the 10-level score; however, it is not surprising to see an improvement with fewer categories, as fewer choices should improve intrarater agreement. By either scale (3-, 4-, or 10-category), these deviations are purported to represent sizeable differences in functional (“performance”) status. The lack of reliability in KPS reporting raises concerns when applying the KPS as a proxy for frailty and a metric to be considered when evaluating candidacy for kidney transplantation.

To abrogate the risk of capturing a real decline in performance status, we needed to limit our analysis to patients who were evaluated at 2 or more programs within a 3-mo period, ergo, the relatively small fraction (2%) of the overall kidney transplant population in our analytic cohort. This limits the generalizability, as multiple listings may not be feasible for most patients. A crude comparison of baseline KPS showed patients with multiple listings at different centers tended to be healthier (mean score = 82 versus 79). They may also be better resourced because traveling to multiple centers can be costly. It is possible that patients with fewer KPS also have lower scores, and these would be more reliable. However, when we relaxed the 3-mo criterion included in the cohort definition, we found that the reliability in the scores declined. This trend indicates that our estimates may overestimate the reliability of the KPS in patients with end-stage kidney disease. Retrospective studies are limited in their capacity to control for unknown factors, and it is possible that these factors might affect the precision and hence the reliability of the KPS. Our results are also limited to transplant candidates and may not reflect the reliability of the KPS in patients not on the waitlist.

In the last decade, there has been an increased proportion of transplant candidates with lower KPS compared with previous years, supporting a growing trend of kidney transplantation in more frail candidates. Moreover, changes in the kidney allocation system in December 2014, which increased the availability of transplantable kidneys to many candidates who previously had more limited access may have contributed to this trend.^20,21^ Although the majority of patients are in the normal performance category, we observed a significant shift from 7% of patients in 2006–2010 in the lower 2 KPS categories to 10% of patients in 2016–2020. Such a shift in the demographics of candidates toward an increasingly frail population supports an increasing need for a reliable estimate of frailty when considering allocation of a scarce resource and the immense benefit that can accrue to successful kidney transplant recipients.

Most studies evaluated the reliability of the KPS in patients with cancer. Chow et al^22^ performed a meta-analysis comparing the reliability of reporting of the KPS, the palliative performance scale and the Eastern Cooperative Oncology Group performance status. They found that the KPS demonstrated the best reliability compared with other measures. Yet, with the exception of only 2 studies, the analysis was performed among patients with advanced or terminal cancer and only 1 of these 2 studies investigated KPS in patients with end-stage kidney disease. The advanced cancer population differs enormously from the kidney transplant candidate population. Patients with advanced cancer often experience substantial degrees of debility and disability. Although patients with advanced chronic kidney disease, including those receiving dialysis, commonly experience functional impairment, generally healthier patients are typically referred for kidney transplant evaluation. In transplant clinics, the KPS is often performed as quick assessments during clinical visits by varying providers (eg, physician, nurse, social worker) with no standardized approach among transplant centers. A recent letter to the editor also suggested that the subjective nature of the KPS makes it prone to bias in patients undergoing liver transplantation.^23^ Bias in the evaluation of kidney transplant candidates is no exception.

Our reliability estimate of 43% in the 4-category score is comparable to the Kappa estimate of 46% in 1979 by Hutchinson et al for a 3-category score for patients receiving hemodialysis.^24^ Hutchinson’s is the only study of which we are aware of that previously considered the interobserver reproducibility of the KPS in patients receiving hemodialysis. Their experiment was performed at 1 center, whereas our study evaluated the use of KPS in practice on a national scale. They found that the proportion of patients with scores in agreement was 71% for the 3-category and 29% for the 10-point score. Comparatively, we found 60% agreement for the 4-category scale and 30% agreement for the 10-point score. Some functional decline over time is expected in candidates on the waitlist, and this may vary depending on the patients’ baseline functional capacity and its trajectory.^19^ Although it is possible that functional decline may have influenced our analysis, the variability we observed between KPS scores is not likely attributable to major change in a patient’s physical function within a span of just 3 mo. Aside from the relatively short-time frame, one would not expect that a patient who might have experienced an acute illness or a sharp decline in health or functional capacity would undergo evaluation and testing and an alternative, often distant transplant center. We did not observe a significant linear trend in the scores over the 3-mo span; however, time was retained in the model to adjust for potential differences in elapsed time between measurements. Given the consistency between our estimates and those reported in previous studies, we believe our values to be an accurate representation of the variation and reliability (or lack thereof) in KPS score reporting nationally.

Some reasons for the poor agreement in the KPS may be due to the subjectivity of the measure, lack of sufficient training among staff, and lack of standardization in implementation across transplant centers.^25^ One study in patients with cancer was able to achieve 97%–100% agreement by providing staff with training and over an hour to several days of interaction to assess the patient.^18^ Still, there is evidence that KPS performance is worse in patients with CKD compared with patients with other comorbidities.^24^ The studies that report higher reliability also typically involve patients with scores in the bottom half (10%–50%) of the scale.^9,18,23^ Some studies show self-assessment to be a convenient measure and reasonable measure among patients who are on the waitlist for kidney transplantation. Examples include the self-reported 10-item Short Form-36 physical functioning questionnaire (SF-36 PF) or a patient self-reported KPS score.^25–27^ However, self-reported measures are also subjective and can be influenced by factors unrelated to physical fitness.

KPS is the only proxy metric of frailty required by the OPTN; however, several centers have considered more objective methods such as the 6-min walk test, the sit-to-stand test and grip strength.^25,28^ Xu et al developed the Liver Frailty Index specific to patients waitlisted for a liver transplant, which includes grip strength, chair stands, and balance. They found that the KPS was not predictive of mortality compared with their more objective measure.^29^ The Fried physical frailty phonotype is based on a combination of subjective (exhaustion) and objective measures (unintentional weight loss, grip strength, walk speed). A score based on the Fried physical frailty phenotype was associated with higher waitlist mortality and lower kidney transplant rate.^30^ Given the importance of frailty in predicting transplant outcomes, transplant centers should adopt less subjective and more reliable measures that can be used for candidate assessment, counseling, and therapeutic measures.

We conclude that the KPS is not a reliable proxy for frailty in kidney transplant candidates because of its high interobserver variability. More reliable and valid measures are needed to optimally risk stratify the transplant candidate population and improve efficiency in kidney allocation. We propose a move toward use of more objective measures when assessing frailty in kidney transplant candidates.

## Supplementary Material


