# Supplementary material for: Karnofsky Performance Score—Failure to Thrive as a Frailty Proxy?
Source: Transplant Direct. 2021 Jun 8;7(7):e708. doi: 10.1097/TXD.0000000000001164 (PMC8191697; doi:10.1097/TXD.0000000000001164)
Supplement: Supplementary file 1 [file txd-7-e708-s001.pdf]

SUPPLEMENTAL MATERIAL

Appendix S1. Description of the random effects models and estimates of agreement.

Appendix S2. Comparison of the linear model and probit model.

Appendix S3. Sensitivity of the ICC estimate to the ascertainment window.

Appendix S1: Description of the random effects models and estimates of agreement.

We assumed *i=1, … , I* measures, *j=1, . . ., J* patients, and *k=1, . . ., K* transplant centers with KPS 10-level scores (Y_{ijk}). To describe the relationship between the scores within the same patient we assumed the following random intercept model[12] ("patient only random effect model") with covariate *X*:

$$Y_{!"\#} = \beta_{\$"} + \beta_{\%}X + e_{!"\#}$$

$$\beta_{\$"} \quad \sim \quad N(\gamma_{\$},$$

$$\tau_{\$\$})\ e_{!"\#} \quad \sim$$

$$N(0, \sigma^{'})$$

where the intercept, $\beta_{\$"}$, was allowed to vary by patient, $\gamma_{\$}$ was the average score for all patients, and $\tau_{\$\$}$ was the variance in the patient mean scores. The distribution of the 10-level scores was approximated by the normal distribution with variance $\sigma^{'}$. By including a random effect in the model, we allowed the total variation to be partitioned into patient ($\tau_{\$\$}$) and residual variation ($\sigma^{'}$) so that we could estimate the intraclass correlation coefficient (ICC) for the patient as [10,12] :

$$ICC = \frac{\tau_{\$\$}}{\tau_{\$\$} + \sigma^{'}}$$

Assuming a normal distribution for KPS scores may not be a reasonable assumption given that most of the scores were between 50 and100 [30]. Hence, we also considered an ordinal model whereby instead of modeling $Y_{!"\#}$ as a continuous outcome we modelled the predicted probability, $\pi 4$, of each of the 4 intervals, *w*: (0, 40), (50, 60), (70), (80-100). The probabilities were transformed into z-scores via a probit link function similar to a logistic regression model [31]:

$$probit[\pi 4(w)] = \beta_{\$"} + \beta_{\%} X$$

$$\beta_{\$"} \quad \sim \quad N(\gamma_{\$}, \tau_{\$\$})$$

In this case the patient level ICC was estimated as [14] :

$$ICC = \frac{\tau^{\$\$}}{\tau_{\$\$} + 1}$$

In addition to the correlation within patients there was also correlation within centers that was addressed by adding a center level random effect to the model. Since the relationship between centers and patients was not hierarchical, we assumed a 2-level crossed design where patient and center have crossed random effects. (see Appendix S1; Figure S1 A). This was modelled as a "patient and center random effects model":

$$Y_{!"\#} = \beta_{\$"\#} + \beta_{\%} X + e_{!"\#}$$

$$\beta_{\$"\#} = \theta_{\$} + b_{\$\$"} + c_{\$\$\#}$$

$$b_{\$\$"} \quad \sim \quad N(0, \tau_{(\$\$})$$

$$c_{\$\$"} \quad \sim \quad N(0, \tau_{)\$\$})$$

$$e_{!"\#} \quad \sim \quad N(0, \sigma^{'})$$

Where $\theta_{\$}$ represents the overall average score, $b_{\$\$"}$ represents the average difference (from the mean) for the scores for patient *j* and $c_{\$\$"}$ represents the average difference for the scores for center *k*. A crossed design allows patients to be associated with multiple centers as opposed to a nested design where all measures for the same patient are nested within a single center. We estimated the correlation (agreement) of scores for the same patient with different centers as [12]:

$$corr\text{A}Y_{!"\#}, Y_{!*"\#*}\text{B} = \frac{\tau_{(\$\$}}{\tau_{(\$\$} + \tau_{)\$\$} + \sigma^{'}}$$

And the correlation (agreement) of scores for the same patient at the same center as:

$$corr\text{A}Y_{!"\#}, Y_{!*"\#}\text{B} = \tau_{(\$\$}\tau_{(}+_{\$\$}\tau+_{)\$\$}\tau_{)}+_{\$\$} \quad \sigma^{'}$$

Most ICC estimates, correlations, and their 95% confidence intervals were estimated using the NLMixed and Mixed procedures in SAS for Windows version 9.4 [14]. We applied the R2Winbugs package [17] in R Studio to confirm our results for the crossed effect model and obtain a 95% credible interval around the ICC estimates.

Figure S1: Example diagram of the structure of correlations between patients and centers where patients have variable number of scores and not all patients are seen at the same centers.

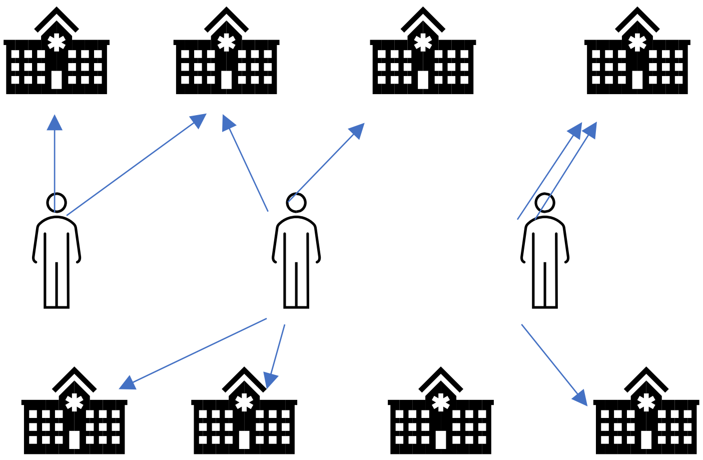

Appendix S2: Comparison of the linear model and probit model.

Table S1: Comparison of the linear model and the ordinal probit model estimate of the intraclass correlation coefficient (95% CI) for the patient random effect‡.

Score Type	Linear model	Ordinal Probit Model
10-level<br>10, 20, 30, 40, 50, 60, 70, 80, 90, 100	30% (28%, 32%)	Could not be estimated.
8-level<br>≤30, 40, 50, 60, 70, 80, 90, 100	30% (28%, 32%)	32% (30%, 34%)
4-level<br>0-40, 50-60, 70, 80-100	30% (28%, 32%)	43% (41%, 46%)
3-level<br>0-40, 50-70, 80-100	36% (34%, 38%)	54% (51%, 57%)

‡Patient only random effects model

Appendix S3: Sensitivity of the intraclass correlation coefficient estimate to the ascertainment window.

Table S2: Percentage of variation explained by the patient intraclass correlation coefficient (ICC)‡ extending the 3-month cohort to 6 and 12 months.

	10-category score		4-category score	
Cohort	Time and year adjusted	Fully adjusted†	Time and year adjusted	Fully adjusted†
3-month cohort (from Table 2) 8,197 candidates, 16,826 KPS	30% (28%, 32%)	23% (21%, 25%)	43% (40%, 46%)	36% (33%, 39%)
6-month cohort 13,215 candidates, 27,536 KPS	25% (24%, 27%)	19% (18%, 21%)	37% (35%, 39%)	29% (27%, 32%)
12-month cohort 21,826 candidates, 46,252 KPS	20% (19%, 22%)	15% (14%, 16%)	31% (30%, 33%)	24% (22%, 26%)

‡Patient only random effects model, patient level ICC (95% CI)

†Adjusted for age, sex, race, ethnicity, comorbidity, dialysis vintage, time, and year.
